# Controlled motion of electrically neutral microparticles by pulsed direct current

**DOI:** 10.1038/srep10162

**Published:** 2015-05-08

**Authors:** Xinfang Zhang, Rongshan Qin

**Affiliations:** 1 Department of Engineering and Innovation, The Open University, Walton Hall, Milton Keynes MK7 6AA, United Kingdom

## Abstract

A controlled motion of electrically neutral microparticles in a conductive liquid at high temperatures has not yet been realized under the uniform direct electric current field. We propose a simple method, which employs pulsed direct current to a conductive liquid metal containing low-conductivity objects at high temperature. The electric current enables the low-conductivity particles to pass from the centre towards the various surfaces of the high-conductivity liquid metal. Most interestingly, the directionality of microparticles can be controlled and their speed can be easily regulated by adjusting pulsed current density. We find that the movement may arise from the configuration of electrical domains which generates a driving force which exceeds the force of gravity and viscous friction. All of these features are of potential benefit in separating the particles of nearly equal density but distinctly different electrical conductivities, and also offer considerable promise for the precise and selective positioning of micro-objects or the controlled motion of minute quantities of surrounding fluids.

Microscopic objects immersed in a fluid, whether at high or low temperatures, must accommodate their motion to effectively high viscous environments. Tremendous effort has been made to explore promising strategies to induce motion of microparticles. Applying an external filed, which can be magnetic^1^,^2^,^3^,^4^,^5^, electric^6^,^7^,^8^ and UV light^9^, is one way to generate motion. Of these, electric filed is especially convenient for practical purposes since their magnitudes, phases, and frequencies can easily to be adjusted. The motion of polarized particles in non-uniform alternating current (AC) or direct current (DC) electric fields (termed dielectrophoresis) has been studied extensively^10^,^11^,^12^,^13^,^14^, as well as the particle motion induced by the Lorentz force (termed electromagnetophoresis)^15^,^16^,^17^. However, a controlled motion of electrically neutral microparticles in a conductive liquid at high temperatures has not yet been realized under the uniform DC field. Moreover, the precise navigation of microscale objects at high temperature is extremely challenging because of the combination of Brownian motion and strong viscous force^18^. Generally speaking, the most important issue for the actuation of particles is to break the equilibrium state of the object. Energy injection is therefore a prerequisite for initiating particles movement. This movement must be continuous and not time reversible. Recently, a rapidly expanding subdomain (such as metallurgy) is the request for microparticles below 10 μm able to move in high-temperature fluids which appear viscous. Electromagnetic stirring technique is generally applicable for separation of the particles above 50 μm in diameter, accompanied by significant energy consumption and high cost^19^. For these reasons, particular importance has been given to develop a novel green processing method for manipulation of micro/nano particles in the melts. Usually, oxides and sulphides have higher electrical resistivity (lower conductivity) than that of the metal; thus the locations of the particles in the suspension system will affect the distribution of electric current^15^,^20^. It can be seen from Fig. 1, the current distribution will be uniform in the case of a sphere with conductivity equal to that of the surroundings (Fig. 1a). In contrast, the distribution is markedly different when the conductivity of a sphere is lower than the environment (Fig. 1b). Electrical processes on the surface of the sphere tend to change the current distribution from the one pictured in Fig. 1b to that of Fig. 1a. This is driven by the tendency to reduce the system free energy. The effect would be greatest in the region of the highest current density. Therefore, the change of system free energy induced by different current distributions may expel a high resistivity object from a low resistivity matrix.

Here in the present work, a uniform pulsed direct current^21^,^22^,^23^,^24^, has been implemented to actuate the micrometer-sized Al_2_O_3_ and MnS particles (<10 μm) in a molten metal (>1450 ^o^C). Application of pulsed electric current has a particularly large advantage in two ways, one is that it is especially convenient for practical purposes since its magnitude, phase, duration, and frequency can easily be adjusted, and the second is that the consumed electric power in electric current pulse processing was 0.001 Watts, which was much less than the power of a household fluorescent light.

## Results

### Influence of electric current on Al_2_O_3_ particles distribution

The distributions of the Al_2_O_3_ particles across the samples are as follows. For the untreated liquid (Fig. 2a), these particles (over 260 features) with various sizes were randomly dispersed in the matrix. When the liquid was treated with electric current, the particles were no longer uniformly dispersed in the matrix (Fig. 2b); they were found to gather around the surface and disappeared from the inner part of the matrix. It can be seen from Fig. 2b, there are virtually no particles near the centre line. However, the number of particles increased in the regions close to the surfaces (*e.g.* top surface and bottom surface). In order to characterize the overall distribution of particles across the samples (Fig. 2c), the number of particles per unit area is drawn with the distance from the centre. It can be seen from the plot, the number of particles was distributed, more or less, uniformly across the sample where no electric current was applied. However, in electrically pulsed samples the particles in the middle area of the sample have nearly all been pushed away by the electric current. In the area close to the surface, the number of particles achieved a local maximum. This indicates that the electric current causes particles in liquid metal to move toward the surface. Another significant observation is that the particle population changes with distance; there are three types, namely (i) steady and unchanged (0 < distance < 2.5 mm), (ii) approximate monotonically increasing (2.5 mm < distance < 5 mm), (iii) approximate monotonically decreasing (5 mm < distance < 7.5 mm).

### Size distribution of the Al_2_O_3_ particles

We have also examined the size distribution of particles on further statistical analysis by means of histograms, which can give an even closer look, at the surface (Fig. 2e) and inner regions (Fig. 2f) where these particles separate. The Al_2_O_3_ particles were observed to exhibit a wide variation in sizes (0.5 to 11.5 μm) under various test conditions (Fig. 2d-f), but the average size of the total particles was calculated to be 2.5 μm. This indicated that particles were pushed forward but particle agglomeration did not occur. The analysis also indicated that the Al_2_O_3_ particles, of a size smaller than 1 μm, can be moved from molten metal by the electric current. As mentioned above, conventional methods are used to move particles with diameters >50 μm, but with limited, and controversial separation efficiency^19^. Furthermore, conventional techniques are accompanied by significant energy consumption and high cost. Thus the efficiency (size separation <1 μm or even less) for the impurity motion and the power consumption (0.001 Watts) for the electropulsing technique are far superior to the conventional methods.

### Influence of electric current on MnS particles distribution

Another typical example is the controlled motion of MnS particles in molten metal. The number distributions of MnS for both untreated- and treated-liquids are compared in Fig. 3a,b. In total, more than 3000 features (ranged from 500 nm to 15 μm) across the investigated area were recorded. It was obvious that MnS was driven by the electric current, *i.e*. the particle is expelled towards the surface of the matrix. Fig. 3c shows the population distribution of the MnS particles as a function of distance from the centre. The particles have been pushed towards the surface and the population density is much greater near the surface. In contrast, the MnS keeps a random distribution in the untreated-liquid. Similarly, the population density changes with distance can also be divided into three groups. Furthermore, the electropulsing technique can separate MnS particles >2 μm from the liquid (Supplementary Fig. S1).

### Newly-formed particles

It also can be seen from Fig. 3b, there are some tiny particles near the centre but for several large inclusion remain. The small particles form a pattern in the interior of the matrix. Further SEM analysis was carried out to confirm both the size and location of the particles. The size of the globular particle is approximately 730 nm in diameter. Three particles were distributed near the grain boundaries, and the other three were around the voids (Fig. 3d). Examination with SEM-EDS has been conducted on the particle chemistry. The typical result is shown in Supplementary Fig. S2, indicating that the particles are rich in manganese and sulphur elements. The relative proportion of the major elements varied slightly from particle to particle but they are distributed quite uniformly throughout the particle. Therefore, it is assumed that the particle is a pure MnS. It seems that these particles had nucleated recently and formed during the solidification. In a previous study^25^, it said that the MnS precipitated at grain boundaries, the morphologies of which were spherical or close to the spherical shape and the size of MnS precipitates ranged from 30 nm to 100 nm. The sulphur and manganese contents were relatively high in this study; thus manganese and sulphur may produce new particles during solidification and the newly-formed particles will grow in the furnace-cooling (otherwise, the size of the inclusion already present in the sample is >1 μm). Thus, the newly-formed particles will be held in the interior of the matrix because the melt is transformed into semisolid state and viscous force is increased drastically. In the experiment, electric current is only applied to the molten metal. The power was switched off during solidification. In addition, some large particles near the centreline were also found. In the following section, we discuss why large particles were also distributed in the inner part of the matrix.

## Discussion

**Figure 1:**
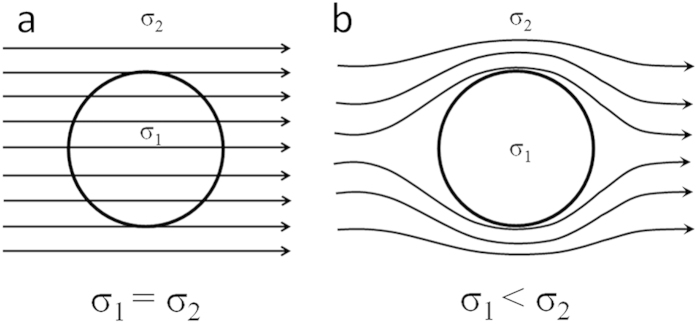
Refraction of electric current lines. Electric current distribution for a sphere with conductivity equal to that of its environment in Fig. 1a, and for a sphere of lower conductivity in Fig. 1b. σ_1_ is the conductivity of sphere, and σ_2_ is the conductivity of the matrix.

**Figure 2:**
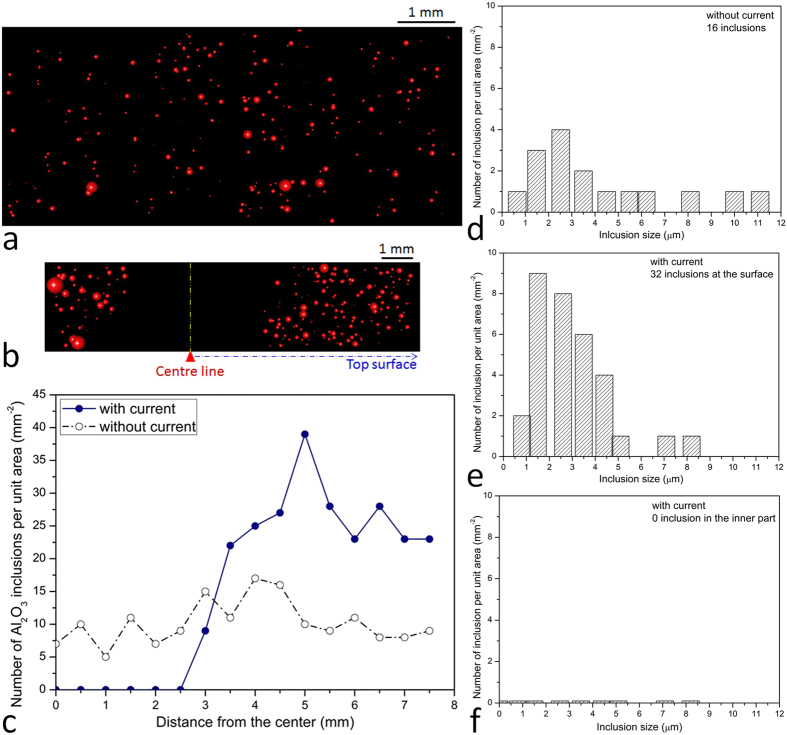
The size and number distributions of Al_2_O_3_ particles in the untreated- and treated-liquids. Automated particle analysis by FEG-SEM was applied to detect the distribution of Al_2_O_3_ particles in the untreated liquid and electric-current-treated liquid. (**a**) Untreated liquid, (**b**) Electric-current-treated liquid, and (**c**) Number distribution of particles in liquid metal without and with electric current treatment. (**d**) Particle size in the sample without electric current treatment, (**e**, **f**) After treated by electric current, the particles are dispersed at the surface with the varied sizes. But they disappear from the inner part of the matrix. The average size of the total particles is calculated to be 2.5 μm. Here it should be noted that the full length of the sample was approximately 22 mm, but only 15 mm length was scanned here (from the top surface of the sample). Thus, we cannot observe the symmetrical distribution of particles on the top and bottom surface, although this is the true case. The uncertainty associated with each particle number is ±3. The relative size of the sphere in (**a**) and (**b**) characterizes the actual size distribution of the particle in (**d**-**f**).

**Figure 3:**
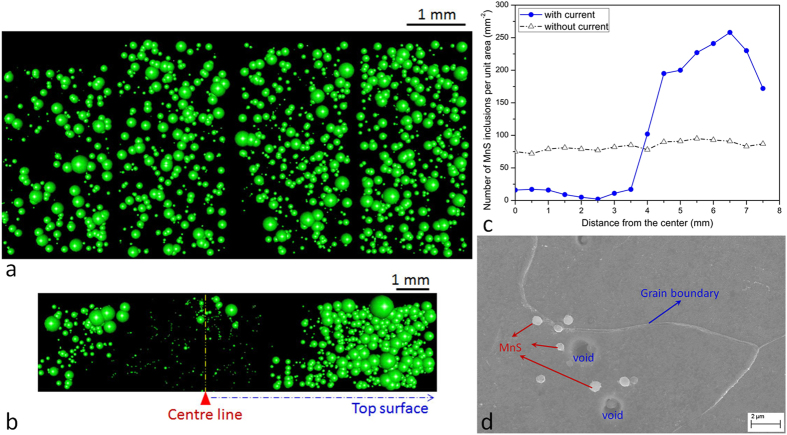
The distributions of MnS particles in the untreated- and treated-liquids. The characterization of newly-formed particles by SEM. Automated particle analysis by FEG-SEM was applied to detect the distribution of MnS inclusions in the untreated liquid and electric-current-treated liquid. (**a**) Untreated liquid, (**b**) Electric-current-treated liquid, and (**c**) Number distribution of particles in liquid metal without and with electric current treatment. (**d**) SEM image showing the morphology and size of MnS in the inner part of the matrix. The globular particles nucleated near the grain boundary or voids. It size is about 730 nm. The uncertainty in values of the particle number for each datum point is ±3. The relative size of the sphere in (**a**) and (**b**) characterizes the actual size distribution of the particle in the Supplementary Figure S1.

A possible mechanism for the observed migration is given in the following sections. The application of an electric current alters the system free energy which is changed with the distribution of particles, as shown in Fig. 1. When the particle moves from one position *d* to another position *d* + Δ*d*, the change of the system free energy may produce a driving force in the perpendicular direction to electric current. This driving force will lead to a directional motion of the objects. Based on numerical calculations, the physics of the motion of the particles can be described by the equations briefly presented in the Supplementary Information. The calculated equivalent driving force from the electric current to the electrically neutral particle can be divided into three zones, namely pushing-trapping-expelling^23^; while the number of particles as a function of distance in Fig. 2c and 3c also exhibits the corresponding change. An approximate expression for the force derived for the effect of the electric curren^23^


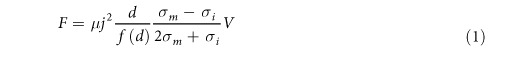


where σ_*m*_ is the electrical conductivity of liquid matrix, σ_*i*_ is the electrical conductivity of the particle, *j* is the current density, *μ* is the magnetic permeability, *V* is the volume of spherical particle. *f* (*d*) increases monotonically but nonlinearly as *d* increases. From a mathematical point of view, if *d* = 0, then *F* = 0. It can be inferred that a small number of particles in the vicinity of the centre line cannot be driven. The distribution of MnS particles in Fig. 3c near the centre line indeed confirms the rationality of the Eq. (1).

**Figure 4:**
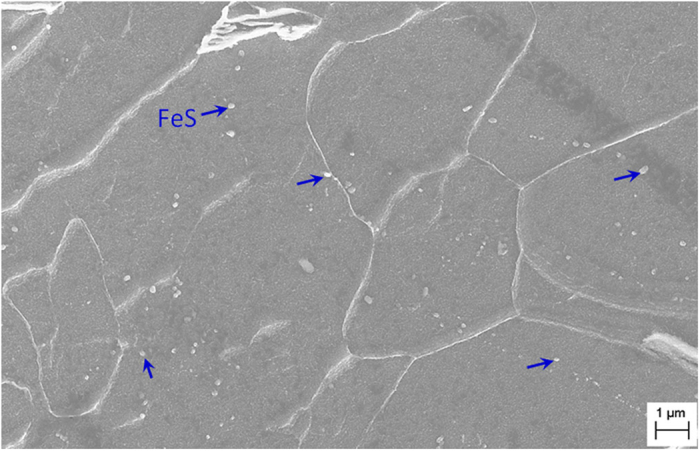
SEM image showing the morphology and distribution of FeS in the inner part of the matrix.

In the expression of the force, it is known that the key factor in the motion of particles is the difference in electrical conductivities between the particle and the metal matrix. In general, the conductivity of the liquid has a value of 10^5^ Ω^-1^m^-1^ and that of Al_2_O_3_ above 1673 K is approximately 10^-2^ Ω^-1^m^-1^. Thus the value for Al_2_O_3_ particles is 10^7^ times smaller than that of the liquid. For MnS, the value is 10^-2^ Ω^-1^m^-1^ above 1273 K which is 10^3^ times smaller than that of the liquid. In addition to Al_2_O_3_ and MnS, there are other types of particles in the metal, such as ZrO_2_, CeO_2_, Cr_2_O_3_, and Ti_2_O_3_. Their conductivities are also much smaller than that of the liquid. Electrical conductivities of various types of particles in the metal are listed in Supplementary Table S1. On the basis of Eq. (1), it can conclude that these particles will also be driven from liquid by the electric current. In addition, there are some important phases with good conductivities in the metal, such as MX, M_23_C_6_, and FeS. These phases should be used to verify the validity of the mechanism. But these phases cannot survive in the liquid because of their low decomposition temperatures^26^,^27^,^28^. Figure 4 shows a uniform distribution of FeS in the electric-current-treated liquid. As demonstrated by the arrows in Fig. 4, some particles are in the inner part of the matrix and others are on the grain boundaries.

Finally, we turn to the question: how to keep the microparticles going in a steady direction and to steer and navigate them towards their destination by temporal and spatial regulation of the speed? The answer can be obtained from the derived expression of particle speed under pulsed electric current (the equations briefly presented in the Supplementary Information). According to the calculated values, the velocity of the particles with electric current is proportional to particle volume, pulsed current density, and the particle position, while that of particles without electric current is only related to their sizes. When the particles are located at the centre of the matrix, the same velocities are obtained for the untreated and treated particles controlled by gravitational force, buoyant force and viscous force. Once the particle is disturbed from the centre, it will be pushed quickly toward the surface by the pulsed electric current. Furthermore, the objects can be driven to move toward other desirable directions by simply adjusting the arrangement of electrodes.

In our experiment, we present a new concept for the controlled motion of electrically neutral objects with sizes ranging from submicron to micron. The realization of controllable microparticles motion by pulsed direct current is far superior to electromagnetic-based method in view of the separation efficiency, power consumption, and cost. A driving force generated under electric current in the small scale to overcome the drag force exerted by the surrounding liquid. Owing to the direction and speed can be regulated, this innovative method might possibly be useful in the precise and selective positioning of micro-objects or the controlled motion of minute quantities of surrounding fluids as well as the application of particle separation.

## Methods

### Particles

A metal containing MnS particles was selected for the study. The metal was placed in a graphite crucible and heated in an induction furnace. Once the metal was molten, aluminum wire was added into the liquid to produce Al_2_O_3_ particles.

### Materials

The chemical compositions (wt.%) of both the investigated metal and the metal electrode were listed in Supplementary Table S2. In order to confirm the difference in melting point, the phase diagrams of both metals were calculated using Thermo-Calc software. From the calculated diagrams, the melting temperature of the liquid metal at 1469 ^o^C is lower than that of the metal bar at 1520 ^o^C. Therefore, it seems feasible that the electrode will remain solid in molten metal and this requires careful adjustment of the output power of the induction furnace. Since the metal bar does not melt, the Al_2_O_3_ and MnS particles (melting point: 2072 ^o^C and 1610 ^o^C, respectively) will remain solid in molten metal.

### Pulsed experiment

In order to apply the electric current into the liquid, a pair of metal bars was submerged into the liquid to act as the electrodes. In order to determine the effect of electric current, two sets of experiments were carried out, one without electric current and the other with electric current. The experimental procedures were identical except for the application of electric current. The microstructures of the two sets of samples determine the effects of the electric current treatment. In the blank experiments the electrodes were submerged in the liquid without connecting the power. In the second set of experiments the sample was treated with the electric current. The process is illustrated schematically in Supplementary Fig. S3. The direction of the applied electric current, the current-inducted magnetic field and the possible direction of migration of the particles are indicated in the figure. Pulsed electric current rather than continuous electric current was applied to prevent Ohmic heating. The pulse was applied in square-wave form. The frequency of the pulse was 1 Hz and the duration of each pulse was 60 μs. The density of the pulsed electric current was 1.6×10^6^ A/m^2^. The total treatment time was 8 minutes. The length of the investigated sample is 0.022 m. No skin effect was noted in the experimental analysis. This is attributed to two reasons: (a) The sample was set in a gap between electrodes. The aspect ratio between longitudinal and transverse dimensions of sample is almost 1. The electric current flow behavior in the sample is mainly affected by the inlet and outlet boundary conditions and is an unestablished flow. The conventional equation for calculation of the skin effect is valid only for the established flow. (b) The shape of the wave is square with on-load and off-load duration vastly heterogeneous.

### Characterization

The longitudinal section was observed to detect the distribution of the particles. The cross-section was not examined because of the symmetry of the pulse processing along the direction. The furnace-cooled samples were longitudinally sectioned and polished for metallographic examination. The composition of the particles in the matrix was analyzed with a scanning electron microscope (SEM) using a microscope equipped for energy-dispersive spectroscopy (EDS). The distribution of the particles across the sample was examined by automated inclusion analysis using field emission gun-scanning electron microscope (FEG-SEM). This technique combines the advantages of EDS with that of digital image analysis of backscattered electron micrographs. It provides fast measurements of composition, size and morphology for thousands of particles embedded in the matrix, giving a more statistically-sound particle size distribution in the sample compared with manual particle size measurements.

### Software

The collected data were analyzed and plotted using in-house developed MatVisual software.

## Author Contributions

X.F.Z. performed experiments, analysed data, and wrote the paper, R.S.Q. planned the research, analysed data, and edited the paper. These authors contributed equally to this work.

## Additional Information

**How to cite this article**: Zhang, X.F. and Qin, R.S. Controlled motion of electrically neutral microparticles by pulsed direct current. *Sci. Rep.* **5**, 10162; doi: 10.1038/srep10162 (2015).

## Supplementary Material

Supplementary Information
