# Supplementary material for: Controlled motion of electrically neutral microparticles by pulsed direct current
Source: Sci Rep. 2015 May 8;5:10162. doi: 10.1038/srep10162 (PMC4424834; doi:10.1038/srep10162)
Supplement: Supplementary Information [file srep10162-s1.doc]

**Supplementary Information**

**Controlled motion of electrically neutral microparticles by pulsed direct current**

Xinfang Zhang* & Rongshan Qin*

*Department of Materials, Imperial College London, Exhibition Road, London SW7 2AZ, United Kingdom*

**Supplementary Discussion**

**Supplementary Table S1**

**Supplementary Table S2**

**Supplementary Figure S1**

**Supplementary Figure S2**

**Supplementary Figure S3**

**Supplementary Figure S4**

**Supplementary Figure S5**

***Supplementary Discussion***

A force from electric current to electrically neutral microparticles

As mentioned above, the two most important types of particles in metal were moved in molten metal by the application of an electric current; these particles were driven from the interior of the metal to the surface of the metal. However, once nucleation and growth of particles was resumed during solidification, the electric current no longer affects nucleation and the formation of MnS (Main text data Figure 3b and 3d). More importantly, the electropulsing technique works for particles with sizes less than 1 μm and thus has advantages over conventional techniques which can only effectively remove particles of > 50 μm. Furthermore, this current-driven technique requires low power consumption; accordingly its production cost is low. Therefore, understanding the separation mechanism of this technique will greatly help to improve its application in metallurgy and biomedicine where the separation of particles of nearly equal density but distinctly different electrical conductivities.

The numerical calculations carried out in a previous study can be used to derive a possible mechanism for the separation of a particle from liquid. The movement of the particles in a direction perpendicular to that of the electric current is not due to electromigration. This latter force involves momentum transfer from the conducting electrons to the particle. The direction of the electromigration force is the opposite of that of the electric field. It should also be noted that the application of an electric current alters the system free energy which is changed with the distribution of particles, as shown in Main text data Figure 1. When the particle moves from one position $d$ to another position$d+\Delta d$, the change of the system free energy will produce a driving force in the perpendicular direction to electric current. The calculated equivalent driving force from the electric current to the electrically neutral particle can be divided into three zones. For simplicity, $d=0$ implies that the particle is located at the centre of the matrix. The positions, *P* and *E* is denote locations where *0<d<P* and *P<d<E,* respectively. Thus, the value of the calculated force *F* with the distance *d* is expressed as

$\left\{\begin{matrix} F>0 & 0<d<P & pushing zone \\ F<0 & P<d<E & trapping zone \\ F>0 & d>E & \exp elling zone \end{matrix}\right.$ (S1)

In the statistics of Main text data Figure 2c, 3c, the number of particles as a function of distance also exhibits the corresponding change, namely pushing-trapping-expelling. It can also be shown that the number achieves a local minimum at *E*. An approximate expression for the force derived for the effect of the electric current on the electrically neutral particle at pushing zone can be obtained by numerical calculations

$F=\mu j^{2}\frac{d}{f\left(d\right)}\frac{\sigma_{m}-\sigma_{i}}{2\sigma_{m}+\sigma_{i}}V$ (1)

where $\sigma_{m}$is the electrical conductivity of liquid, $\sigma_{i}$is the electrical conductivity of the particle, $j$is the current density, *µ* is the magnetic permeability, $V=\frac{4\pi R^{2}}{3}$ is the volume of spherical particle, *R* is the diameter of a particle. $f(d)$ increases monotonically but nonlinearly as *d* increases. The width of the sample is usually much larger than that of the size of particle. The pushing zone is, therefore in most cases, the dominant area across the matrix. Moreover, from a mathematical point of view, if $d=0$, then $F=0$ according to Eq. (2). It can be inferred that a small number of particles in the vicinity of the centre line cannot be moved. The distribution of MnS particles (above 1 μm) in Main text data Figure 3c near the centre line indeed confirms the rationality of the Equation (1). Although small MnS particles are generated in the inner part of the matrix, these particles cannot grow to particles of 1 μm or more during rapid cooling. Therefore, the size distribution of particles above 1 μm in the inner part of the matrix provides a good interpretation of the self-consistency of proposed mechanism.

Terminal velocity of the particle under electric current

We now turn to the question: What is the terminal velocity of the particle under electric current? Typically, in the absence of electric current, the velocity of the particle is determined by gravitational force, buoyant force and frictional force. According to Stokes’ law, the frictional force *F_f_* is expressed as$F_{f}=6\pi\mu'R\upsilon$. *μˊ* is the dynamic viscosity and *ν* is the particle velocity. According to Archimedes’ principle, the excess force *F_g_* is due to the difference of the weight of the particle and the buoyancy on the particle. It can be written as$F_{g}=(\rho_{m}-\rho_{i})g\frac{4}{3}\pi R^{3}$. *ρ_m_* and *ρ_i_* are the mass density of the metal and the particle, respectively. *g* is the gravitational acceleration. Demanding force balance $F_{g}=F_{f}$ and solving the velocity gives the terminal velocity *ν_1_* as follows.

$\upsilon_{1}=\frac{2}{9}\frac{(\rho_{m}-\rho_{i})}{\mu'}gR^{2}$ (S2)

For the velocity of pushing zone under electric current, it is determined by gravitational force, buoyant force, the force from electric current to the electrically neutral particle, and frictional force. Thus, solving the force balance $F_{g}+F=F_{f}$can give the terminal velocity *ν_2_*. Here, $\frac{\sigma_{m}-\sigma_{i}}{2\sigma_{m}+\sigma_{i}}\approx\frac{1}{2}$due to the large discrepancies of $\sigma_{m}$and$\sigma_{i}$. Thus, we can obtain the velocity *ν_2_* at pushing zone as

(S3)

In this study, except for *d* and *f(d)*, all other parameters are constants. We assume that the particle size $R=5\times10^{-6}m$ according to the average sizes of these particles in this study. The particle size keeps unchanged with distance, which is different from bubble size increase with distance. The others are listed as follows:$\rho_{m}=7\times10^{3} kg\cdot m^{-3}$,$\rho_{i}=4\times10^{3} kg\cdot m^{-3}$, $\mu'=0.0067 kg\cdot m^{-1}\cdot s^{-1}$, $g=9.8 m\cdot s^{-2}$, $\mu=1.25\times10^{-6}H\cdot m^{-1}$, and $j=1.6\times10^{6}A\cdot m^{-2}$. Thus, we can obtain $\upsilon_{1}=2.44\times10^{-5} m\cdot s^{-1}$in the untreated metal. The terminal velocity *ν_2_* at the pushing zone for the treated metal is calculated to be $\upsilon_{2}=2.44\times10^{-5}+1.33\times10^{-3}\frac{d}{f(d)}$ . According to the calculated values, the velocities of particles in the investigated metals are the function of$\frac{d}{f(d)}$, as demonstrated in Supplementary Fig. S4. The conclusion can be drawn that the velocity of particles with electric current is proportional to$\frac{d}{f(d)}$, while that of particles without electric current does not change over$\frac{d}{f(d)}$. When the$d=0$, e.g., near the centre line, the same velocities are obtained for the untreated and treated particles controlled by gravitational force, buoyant force and frictional force. Once the particle is disturbed from the centre line, it will be pushed quickly toward the surface.

**Supplementary Table S1**

| **Phase** | **Electrical conductivity (Ω^−1^m^−1^) above 1273 K** |
| --- | --- |
| **Molten metal** | 10^5^ |
| **Al_2_O_3_** | 10^−2^ |
| **MnS** | 10^2^ |
| **ZrO_2_** | 10^−1^ |
| **CeO_2_** | 10^−2^ |
| **Cr_2_O_3_** | 10 |
| **Ti_2_O_3_** | 10^2^ |
| **FeS** | 10^5^ |
| **MX** | 10^6^ |
| **M_23_C_6_** | 10^5^ |

**SUPPLEMENTARY TABLE S1** Electrical conductivities of various types of phases in the metal

**Supplementary Table S2**

| **Sample** | **C** | **Si** | **Mn** | **S** | **Cr** | **Ni** | **Cu** | **Fe** |
| --- | --- | --- | --- | --- | --- | --- | --- | --- |
| **Metal** | 0.74 | 0.27 | 0.99 | 0.019 | 0.016 | 0.015 | 0.008 | Bal. |
| **Electrode** | 0.12 | 0.17 | 0.53 | 0.021 | 0.075 | 0.152 | 0.494 | Bal. |

**SUPPLEMENTARY TABLE S2** Chemical compositions (wt.%) of investigated metal and metal electrode

**Supplementary Figure S1**


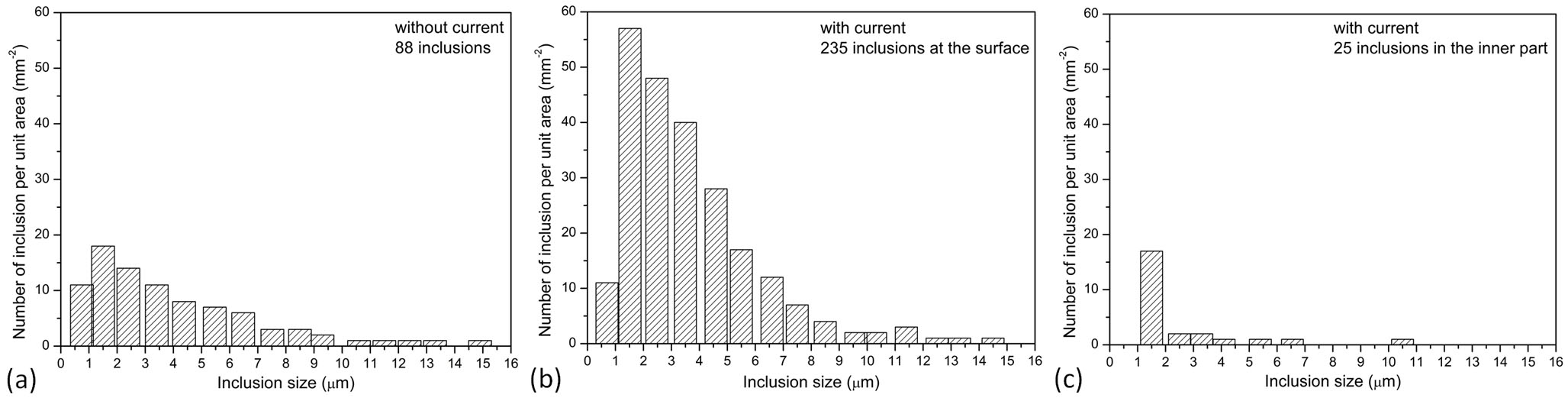


**SUPPLEMENTARY FIGURE S1** Size distributions of MnS particles in liquid metal without and with electric current treatment. (a) Particle size in the sample without electric current treatment. (b, c) After treated by electric current, the particles are dispersed at the surface with the varied sizes. But they disappear from the inner part of the matrix. The average size of the total particles is calculated to be 3.4 μm.

**Supplementary Figure S2**

**
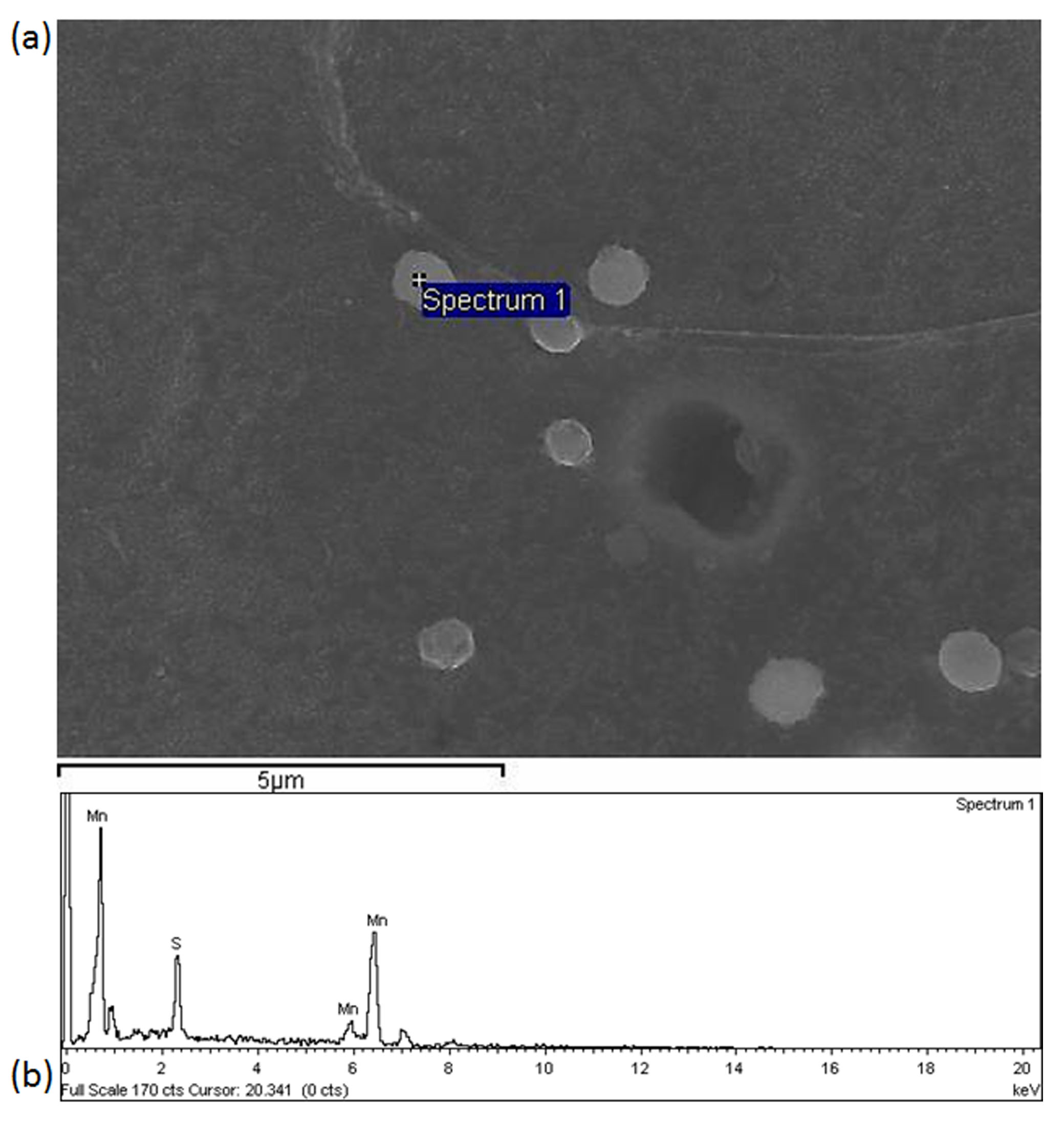
**

**SUPPLEMENTARY FIGURE S2** (a) SEM image showing the morphology and size of MnS (bright phase) in the inner part of the matrix. The globular particles nucleated near the grain boundary or voids. It size is about 730 nm. (b) EDS pattern of the MnS particle.

**Supplementary Figure S3**


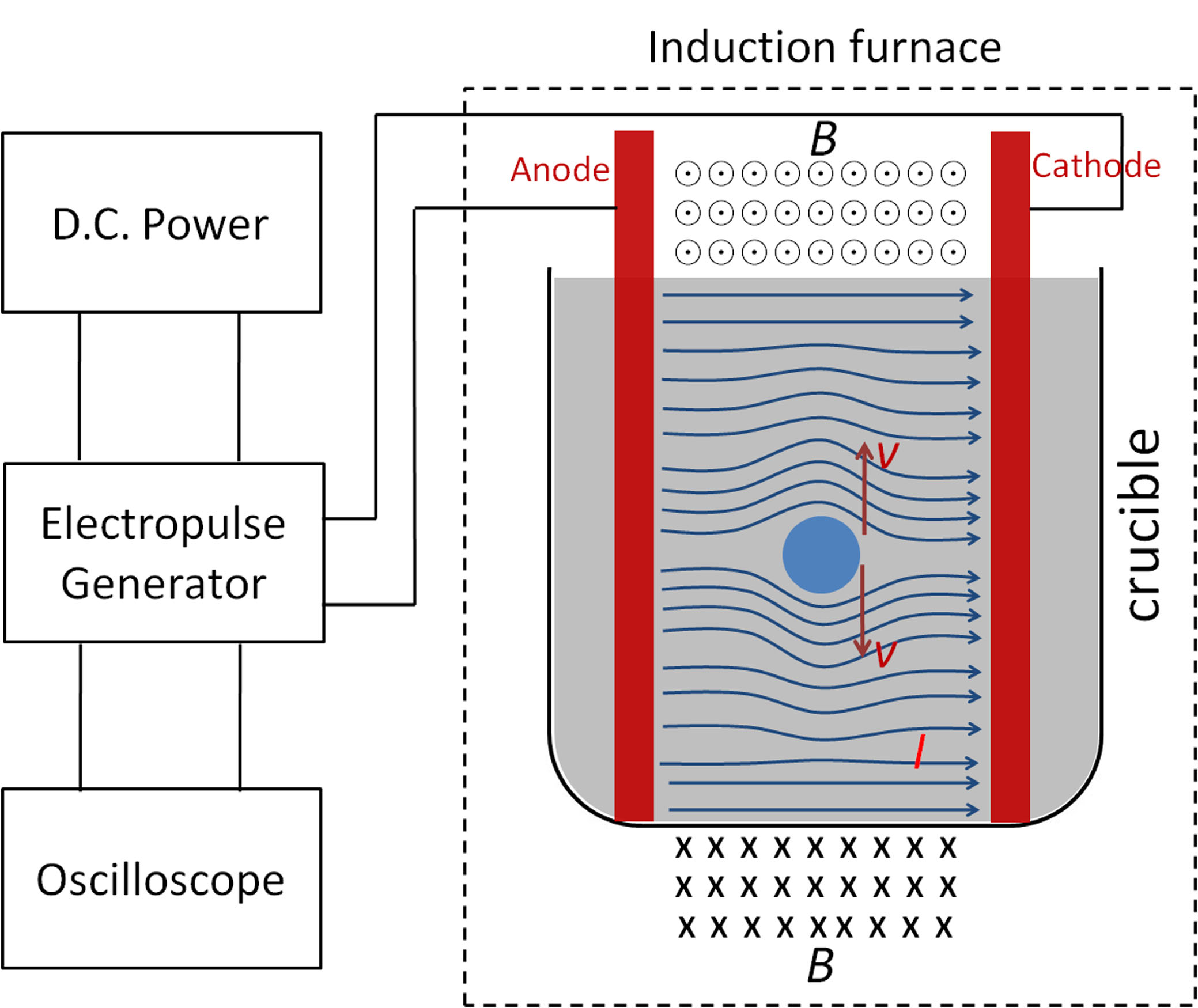


**SUPPLEMENTARY FIGURE S3** Experimental setup for pulsed electric current experiments. *B* is magnetic field, *I* is electric current, and *v* is the migration of the particle.

**Supplementary Figure S4**

**
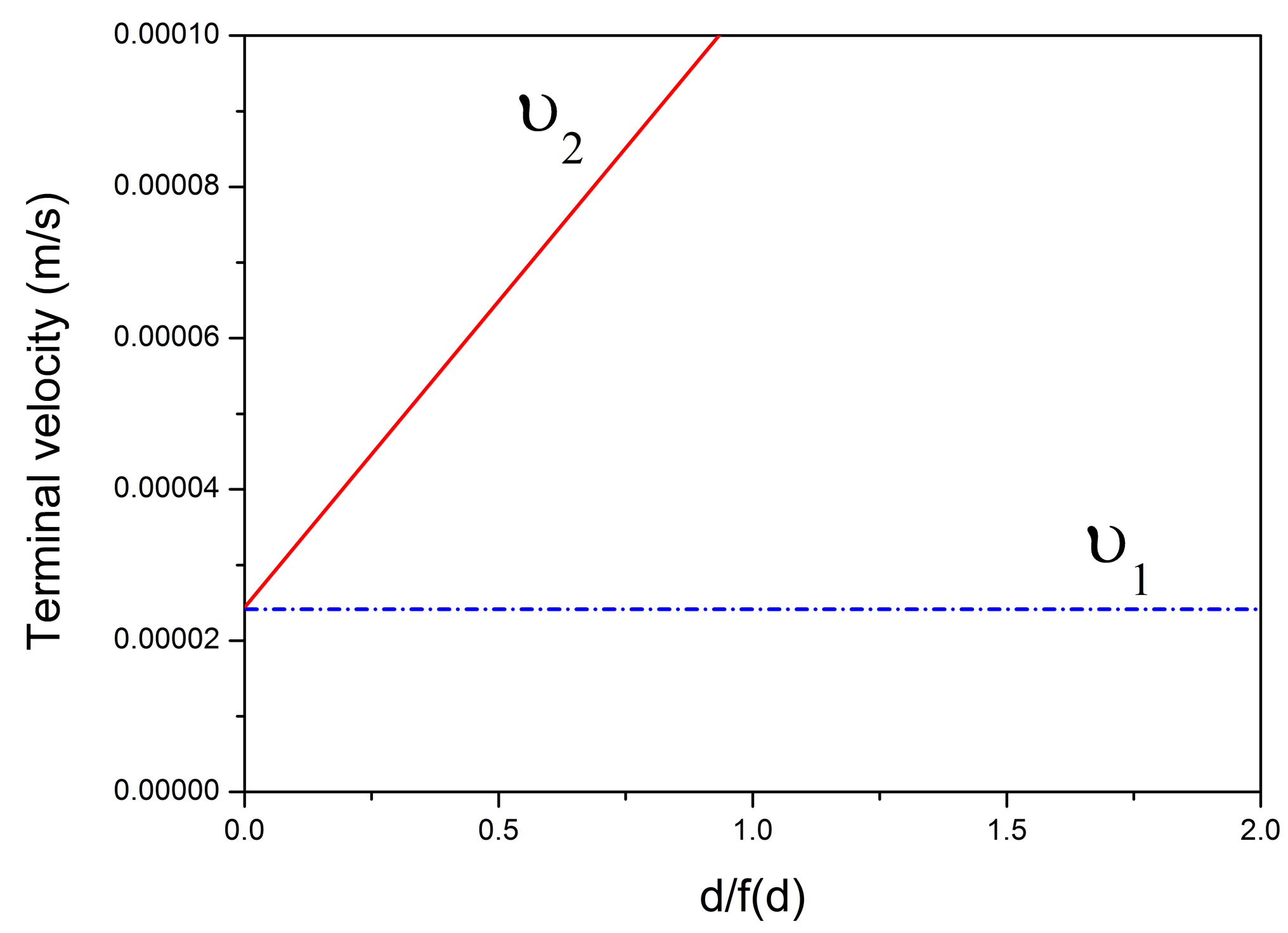
**

**SUPPLEMENTARY FIGURE S4** The change of terminal velocity vs *d/f(d)* for particles in the untreated and treated metals.

**Supplementary Figure S5**

**
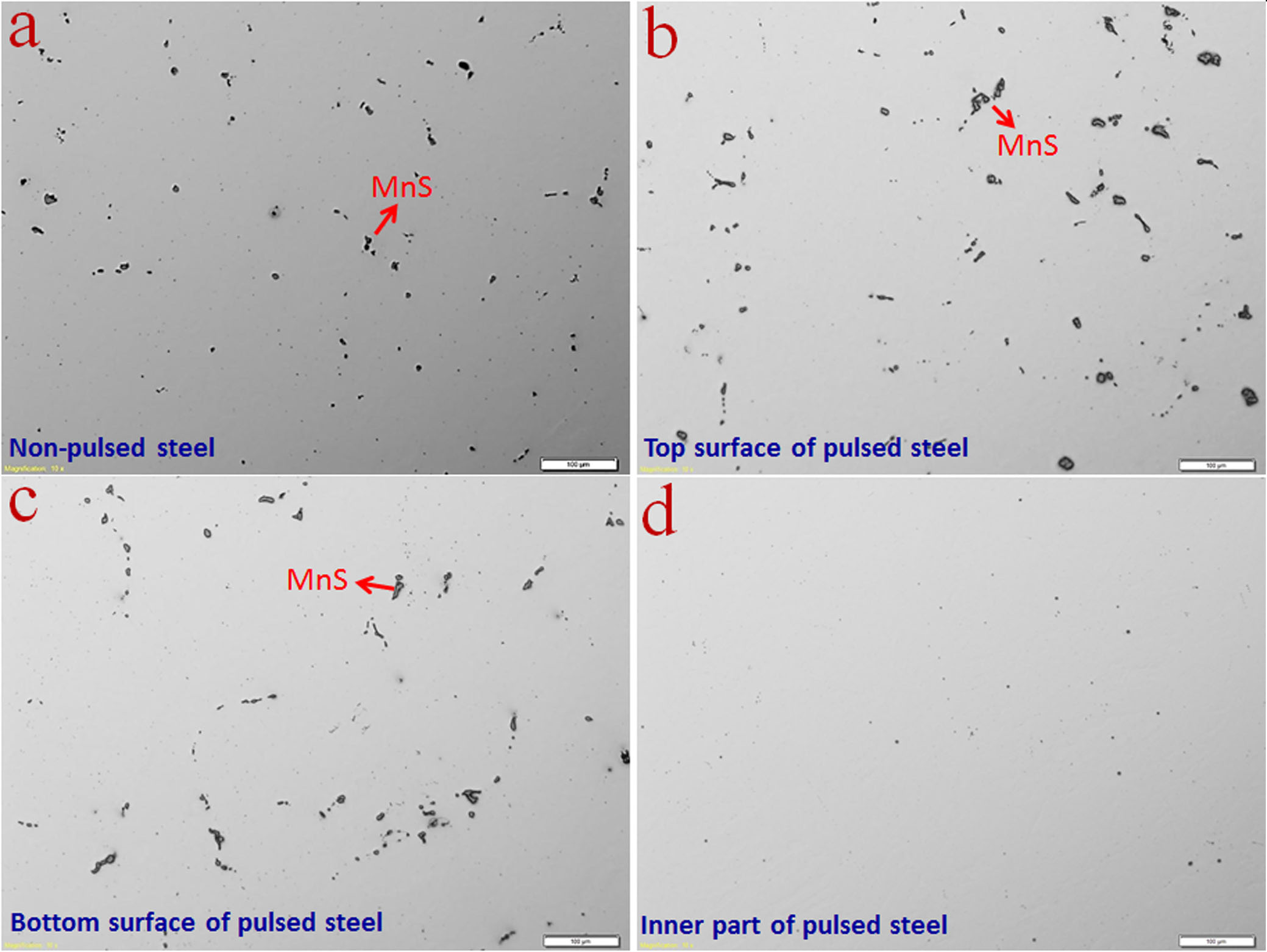
**

**SUPPLEMENTARY FIGURE S5** Distribution of MnS (dark phase) in the metals. (a) Random distribution in untreated as-solidified metal; (b) Large concentration of particles on the top surface in the pulsed metal; (c) Large concentration of particles on the bottom surface in the pulsed metal; and (d) Disappearance of particle from the inner part of the pulsed metal.
